# The Relation of Dental Sepsis to Other Pathological Lesions*Paper read before the Liverpool and District Odontological Society; reprinted from *The British Dental Record*, July, 1921.

**Published:** 1921-08

**Authors:** Frank Colyer

**Affiliations:** K.B.E.


### THE RELATION OF DENTAL SEPSIS TO OTHER PATHOLOGICAL LESIONS *

BY SIR FRANK COLYER, K.B.E., F.R.C.S.

Scarcely twenty years have passed since Dr. W. Hunter drew special attention to the fact that oral sepsis may be and frequently is an important factor in the development of morbid processes in other parts of the body.

Those who were in practice at that time will remember the skepticism with which Dr. Hunter's views were received by the medical and dental professions generally. Fortunately, however, there were a few practitioners who, perceiving the reasonableness of Hunter's contentions, pursued inquiries on the lines which he indicated, and by careful clinical methods, aided by the use of radiograms, gradually accumulated evidence which placed beyond doubt the accuracy of his deductions.

Today dental sepsis as a cause of ill-health is regarded as an established fact, but while recognizing this fact, we must be on our guard to place it in its correct relative position in the group of etiological factors which play a part in the production of disease.

We shall be in a more favorable position to do this if we remember that dental sepsis is not a specific disease, but is a convenient term used to embrace various chronic septic infective processes about the teeth, processes which conform to the same laws which govern pathological conditions in general. To these laws I do not intend making any reference beyond directing your attention to the fact that directly tissues are threatened with infection the body mobilizes its defenses, which are partly local, namely, the raising of a barrier around the infected area, and partly general, namely, the setting up of certain processes to deal

* Paper read before the Liverpool and District Odontological Society; reprinted from *The British Dental Record*, July, 1921.

with the infection should it gain access to the general blood stream.

In lesions of the periodontal membrane the invasion is usually slow and the barrier efficient, at any rate in the early stages, but the defenses may give way directly the general resistance of the tissues is lowered under the strain produced by some other illness or by traumatism. Then the organisms or the toxins, no longer held in check, may start pathological processes in other parts.

Before leaving this question of the defense of the tissues, I wish to dwell for a few moments on the protection of the periodontal membrane from infection. We have no very satisfactory evidence how this is accomplished. The free margin of gum may protect the dental ligament from injury during the process of mastication, and I think the gingival trough probably plays a part in warding off infection.

W. J. Penfold, working in the pathological laboratory of the Royal Dental Hospital, demonstrated that the base of the trough in healthy mouths was usually sterile. It has also been shown that the so-called salivary corpuscles have their origin from the tissues forming the gingival margin, and Mendel has shown that these cells, when present in the gingival trough, are acting as phagocytes. These facts seem to point to a machinery for keeping the trough free from organisms and preventing infection of the deeper tissues.

The work of Noyes and Dewey on the lymphatics of this region is of particular import on this question of infection. These authors have shown that the lymphatics on the inner surface of the gum margin (the surface assisting in the formation of the trough) course directly through the periodontal membrane to the apical region, there to be joined by those from the pulp; from here the lymph vessels find their way to "the mandibular and infra-orbital canals, which they leave in company with the blood vessels and nerves at the mental and infra-orbital foraminæ, emptying eventually into the deep cervical glands and submaxillary glands.'' I am particularly interested in this work of Noyes and Dewey, because it confirms a view I expressed in an early paper on periodontal disease about the paths of infection in dental sepsis. The case which drew my attention to this phase of dental sepsis was as follows:

A female, aged thirty, came under treatment one January for a general gingivitis; there was but slight recession of the gum margins; moderately deep pockets were present around the teeth, and the gums bled freely on the slightest touch. She was a mouth-breather, and beyond the fact that she occasionally suffered from indigestion, there was no apparent symptom of ill-health. Local treatment was advised and faithfully carried out by the patient. In October, she developed suspicions of phthisis, and was treated in a home. When seen in the following June the mouth condition was much worse and the pre-molars and molars were removed. Early in September the incisors and canines were extracted, and in one month there was a gain in weight of eight pounds; she has remained well since that time. The condition of the bone just previous to the removal of the teeth showed that there was only slight bone destruction and no apparent invasion of the medulla from the sockets, the lamina dura being present. The teeth, however, were most instructive. The periosteum covering the roots appeared normal except at the apices, where there were definite signs of trouble as shown by absorption of the tissue and marked translucency of the tissues.

Here, then, we seem to have infection in the gingival trough passing direct to tissues around the apices of the teeth and an infection of sufficient intensity to cause a marked ''reaction.'' This reaction, to my mind, suggests that the soft tissues around the apex of the tooth may possess a protective mechanism with the view of intercepting or neutralizing the passage of infection to the blood stream—acting similarly to a lymph node, but like other defenses, breaking down when submitted to too great a strain. The relation of the defense to the attack is of paramount importance in the development of all infective processes.

With these brief introductory remarks we may now pass to a consideration of how dental sepsis causes or influences other pathological processes.

Dr. Hunter, in 1906, drew attention to the fact that dental sepsis adds to the severity of an attack of scarlet fever, and other writers have shown that enlargement of the tonsil frequently decreases with the removal of carious teeth. Most laryngologists are agreed that dental sepsis is responsible for many cases of chronic pharyngitis and laryngitis, the teeth most to blame are the mandibular molars, especially the third; the deep pocket which is so often present behind this tooth is a happy breeding ground for organisms, and one can frequently trace a definite track of inflamed tissue from the pocket across the pillars of the fauces. In chronic superficial glossitis we have another condition in the development of which dental sepsis plays an important part. Alcohol, syphilis and excessive smoking have been indicted as the three main causes of this condition; to these must be added dental sepsis. I have carefully observed these cases during the last fifteen years, and feel convinced that if the teeth are healthy, alcohol, syphilis and smoking do not alone produce that stage which is known by the term leucoplakia; an advance to this condition only occurs when dental sepsis is present. You may stop the alcohol, prescribe anti-syphilitic remedies and stop the smoking, but if you do not remove the sepsis the disease will progress and probably terminate in epithelioma. To my mind the radical removal of the sepsis is imperative. Not only do I consider that dental sepsis is an important factor in cancer of the tongue, but I think that it has a close causal relationship to epithelioma starting in the gums and the buccal surfaces of the cheeks, for in nearly every case one can obtain a history of long-continued sepsis.

In the foregoing conditions the damage has been to the lining membrane of the mouth, but the effect of sepsis on bone also demands attention. I have seen three cases of myeloma in which there was strong evidence that the dental sepsis was the determining cause. There is reason to think that some of the cases of so-called leontiasis ossea are in reality slow infective processes having their origin in the teeth. Support is given to this view by an examination of monkeys' skulls showing enlargement of the jaw bones; this is a subject I hope to deal with fully in a future communication.

I do not think that we have given sufficient attention to septic osteitis as the cause of many of the cases of neuralgia met with in practice. The tendency is to look for a condition affecting the periphery of the nerve and overlook the possibility that the cause may be a direct infection of the nerve sheath. In neuralgia, due to a peripheral cause, such as infection of the nerves of the pulp, the pain tends to "fly" all over the face, while in neuralgia due to infection of the sheath, the pain follows the course of the nerve; a typical example is that seen in the case where the mandibular nerve is implicated in a septic osteitis in the region of the third molar; ask the patient to map out the course of the pain, and he will indicate a line corresponding to the position of the nerve. We should regard this type of neuralgia with deep concern, because there is a danger of the infection spreading along the nerve sheath, and I have seen a case in which the infection, starting in the position of the left third molar, gradually involved the whole of the second and third divisions of the fifth nerve and necessitated the removal of the Gasserian ganglion.

Peter Daniel* has drawn attention to cases of dysphagia with no apparent cause. In five patients referred to him as cases of œsophageal cancer, repeated examinations and the passage of instruments revealed no stricture. All the patients had marked dental sepsis, and with the removal of this the symptoms entirely disappeared. I have myself seen a similar case. This condition can be explained on the lines that sphincters tend to contract when irritated, as is well shown in pyloric spasm. Now, in the œsophagus about the level of the cricoid cartilage there is a sphincter, which was first described by Sir Everard Home and more recently by Professor Keith; it is probable that in the cases mentioned by Daniel contraction of this sphincter is brought about by the presence of the dental sepsis. Time will not permit me to refer to the relation of dental sepsis to gastro-intestinal conditions, much as I should have liked to discuss its possible relation to gastric cancer.

Of the general pathological conditions attributable to dental sepsis, by far the commonest is the one which is clinically designated by the term "general debility." There is a feeling of ill-health, a lack of energy, tiredness at the end of the day's work, a sallow, unhealthy appearance of the skin and frequently a history of gastro-intestinal disturbance. The symptoms are indefinite. This condition must be regarded as the result of the continued absorption of small doses of toxins, and should be classed as a chronic toxæmia. In a milder form the individual complains of "being below par," and there are many people who become so accustomed to this condition as to regard it as normal, and it is not until after the removal of a septic focus that they realize their previous condition was abnormal.

The rapid improvement in health following the removal of dental sepsis in the child and the adult is often remarkable. Good examples of the general results following the systematic removal of septic teeth were seen at the Croydon

---

* *Brit. Med. Journ.*, January 5, 1910, p. 121.

War Hospital. It was part of the routine treatment of the patient to extract not only the teeth connected with the injured area, but also all teeth that were the source of active sepsis. The weights of the patients were kept over definite periods, and it was found that notwithstanding the facts that the majority of the patients were treated with double metal cap-splints fixed and the diet was of a fluid or pappy type, ninety-five per cent. either retained or gained weight under treatment.

In considering the value of this test one must remember that the cases did not include those with loss of tissue requiring plastic work, but even taking this into consideration, the results obtained are strong proof of the part played by the clean mouth in the general nutrition of the individual.

If dental sepsis can affect so profoundly the general metabolism of the body, it is self-evident that it must have a baneful influence on other pathological processes set into activity by trauma, infection, or any other cause. As an example of the influence of dental sepsis on infective processes, we may take that condition known as chronic pulmonary tuberculosis. Here we have an infecting agent being resisted by the defenses of the lung tissues; if the defenses are strong the disease is kept in check, if weak, then the disease becomes active and spreads. Any condition, therefore, which lowers the general tissue resistance must exert an influence on the lung condition. A paper by R. C. Wingfield, dealing with this subject, was published in the *Lancet* (July 18, 1914). He records the results of radical dental treatment in forty-one cases treated in the tuberculosis department of St. Thomas's Hospital. In twenty cases of undoubted pulmonary tuberculosis, one-half showed distinct improvement and the other half no improvement; in twenty-one cases of doubtful pulmonary tuberculosis, fifteen showed distinct improvement. Of the cases of undoubted pulmonary tuberculosis not showing improvement, one died, three were recorded as worse and the remainder unaltered.

In the one that died and in two out of the three recorded as worse, the pulmonary tuberculosis was active. I think these facts are most instructive. The bodily defenses of one who has the disease in an active form are unable to hold the infecting agent in check. The effect of the removal of the teeth is to increase for a period the amount of toxins absorbed into the circulation, and this has the effect of decreasing the local resistance of the lung tissues, with the result that the disease becomes worse. It therefore seems that, in cases where the disease is active, the radical treatment of the dental sepsis should be delayed until a quiescent stage and then should be carried out very gradually.

As an example of the influence on traumatic lesions the following case may be quoted:

A young man injures his left knee at football. The usual remedial measures are tried without success and a chronic synovitis becomes established. Removal of the dental sepsis is followed by complete recovery.

There is evidence that the toxins absorbed from the mouth may in part be eliminated in the various secretions. H. Waller has shown* that failure of the infant to gain weight is frequently associated with dental sepsis in the nursing mother, and that the removal of the affected teeth has been followed by a rapid improvement in the weight of the child. Many of these children vomit the milk after the feed, a fact which points to the milk containing some toxin which the gastric mucosa will not tolerate. If the secretion of the milk can be so affected by mouth toxins, it is possible, nay probable, that they may affect other secretions of the body, including those of the ductless glands. Our knowledge of the relation of the latter to other body processes is as yet in its infancy, but we do know that they are intimately associated with certain abnormal conditions, for example, acromegaly associated with increased pituitary secretion, cretinism with deficient thyroid, and ex-

---

* Dental Diseases in Nursing Women, *Lancet*, November 4, 1916.

ophthalmic goitre with too much thyroid. May not some of these changes in the ductless glands be due to toxic action combined with an inherited cause? I have met with one case in which the dental sepsis seemed markedly to influence the thyroid in a female with exopthalmic goitre. This patient, just before her marriage, developed symptons of this disease; the condition progressed rapidly and was quite unaffected by the usual treatment. There was generalized periodontal disease; this was treated in a radical manner, and from that time the patient made an uninterrupted recovery, and at the present time (twenty years after the commencement of the disease) shows scarcely any degree of exophthalmos.

Much has been written upon the relation of dental sepsis to the joint affections grouped under the term "arthritis deformans." I propose to deal in some detail with these conditions because they afford an instance of the difficulty there is at times in assessing the degree of damage due to dental sepsis.

In the form usually known as polyarthritis the onset may be rapid and the disease may pass into a chronic stage, or the condition may be slow in onset and progressive. In this disease the periarticular tissues are the first to be involved, the cartilages being implicated in the later stages. The disease usually attacks the joints of the hand and foot; the individuals affected are generally under forty years of age, and although more common in women than in men, it is about equally distributed in boys and girls.

It is generally conceded that polyarthritis is bacterial in origin, and Horder considers the infecting organism is either the S. salivarius or the S. fœcalis—both organisms of low virulence.

There is ample evidence that the source of infection is often from the teeth, the rapid improvement and eventual cure in some cases being most striking. For example, a married woman, aged thirty, hobbled into my out-patient department in 1911, having an arthritis involving several of the joints. The teeth remaining were loose. Radiograms showed extensive destruction of the bone and absorption of the roots of the teeth. As far as could be ascertained there were no other sources of sepsis. The teeth were extracted and recovery followed. I have recently heard from this patient; she has remained quite well and has not had any return whatever of the arthritis. In this case it is evident that the infection arose from the teeth.

In many patients there are other sources of infection, and in these cases it is extremely difficult to express a definite opinion upon the relation of the dental sepsis to the arthritic condition. The following is an example: three years since a man aged about forty-two years was sent to me with arthritis attacking the joints of the hands and feet. He had periodontal disease, but there was a fair degree of reparative power in the alveolar process; he was a mouth-breather; there was old tonsillar trouble in addition to symptoms of gastro-intestinal disturbance. Here we have periodontal disease in a large measure attributable to the mouth breathing; the tonsillar condition is increased by the dental sepsis and a vicious circle is formed. The combined dental and tonsillar sepsis aggravate the gastro-intestinal condition, and this in turn affects the general metabolism, including that of the teeth and tonsils, and vicious circle number two is formed. Under such conditions it is impossible, with our present knowledge, to state whether the dental sepsis is or is not the primary cause—but the balance of evidence is rather against than for. Nevertheless, radical treatment of the dental sepsis is necessary before the tonsillar or gastro-intestinal conditions can be dealt with and the patient freed of his sepsis. The teeth were removed a few at a time, and after each operation there was a fair degree of reaction in the joints but little improvement. Removal of the tonsils failed materially to affect the condition, and it was not until a regular course of treatment for the gastro-intestinal condition was carried out that the patient made any headway. Today his disease is checked and there is considerable improvement in the power to use his limbs. In this case the dental sepsis can only be regarded as a contributing cause by influencing the degree of gastro-intestinal sepsis.

In many cases there is a definite family history of rheumatism, which, after discounting the possibility of similarity of environment, suggests some germinal variation. In these patients the joint condition is due to a combination of intrinsic and extrinsic causes, and although it is imperative to free the individual of sepsis, permanent relief can hardly be expected, because other sources of sepsis may light up the condition.

In osteo-arthritis we have a condition the pathology of which is still obscure. By some authorities the joint changes are considered to be degenerative in type, while others maintain they are of bacterial origin. In these cases, even if periodontal trouble is present, it is of the chronic type, and the radiograms of the alveolar process usually show definite sclerosis of the lamina dura, pointing to ample reaction to any toxins affecting the periodontal membrane. I have seen many cases of this type, and there is to my mind no evidence for the assumption that the mouth condition is the etiological factor.

There is ample evidence that dental sepsis influences the course and development of many cases of nervous and mental disorders. The condition we meet with most frequently in practice is that labelled as "nervous breakdown." In most cases there is a history of an undue mental strain and the failure of prolonged rest to effect an improvement. The removal of dental sepsis, if present, is, however, followed by a rapid recovery. In these cases the sepsis weakens the resistance to the mental strain and is a contributing factor, but not the cause; if the individual had not been subjected to the mental strain, the sepsis by itself would not have produced the nervous breakdown.

This question of the relation of oral infections to mental conditions demands our most careful consideration. In the *Journal of Dental Research,* September, 1919, there is a paper by Dr. H. A. Cotton, in which he states that a marked improvement in mental diseases often follows the radical removal of sepsis, be it in the teeth, tonsils or the intestines. The type of case to which he especially refers is that belonging to the dementia praecox group, and he states that in twenty-five per cent. of his cases the teeth were the etiological factor; in another twenty-five per cent. the teeth and tonsils, and in the remaining fifty per cent. the gastro-intestinal canal was involved. Dr. Cotton is, I think, inclined to place too much importance on the sepsis as an etiological factor and underrates the part played by heredity—indeed, he states that it had very little influence in the etiology or in the *prognosis* of mental diseases. In other words, he would have us believe that the sepsis alone is responsible, and that therefore with its removal a cure is effected and the prognosis good as far as the mental condition is concerned. I have read the paper with some care, and have come to the conclusion that the author has not produced sufficient evidence to warrant his deductions.

We can not ignore the considered opinion of such a distinguished neurologist as Sir Frederick Mott, who emphasizes the importance of inborn factors in the production of neuroses and psychoses and certain degenerative conditions of the nervous system which have been designated under the collective term of abrotrophies. The nature of this inborn factor we do not know. It is probably a feeble resistance of the nerve cells to the action of certain toxins, and amongst these toxins we must include those of dental sepsis. The point I wish to stress is that the nerve condition in the majority of cases can not be produced by the dental sepsis alone—the inborn factor must be present. This is a totally different point of view from that expressed by Dr. Cotton.

Since the middle of the last century diseased teeth have been recognized as a source of eye lesions. To the old writers, bacteria and their toxins were unknown, and the eye lesions were considered to be due to neuroses. It is indeed interesting to read Von Stellwag as quoted by Wedl on this subject. He says: "It is a well-known fact that it is by no means an uncommon occurrence for intense irritation of one or other division of the trifacial nerve to lead to hyperæsthesia, and later to hyperæmia and inflammation in the territory of the ciliary nerves." And again: "Hyperæsthesia of the ciliary nerves may also be induced by exfoliation of an alveolar process, abscesses on the roots of a tooth and the impaction of foreign bodies on an alveolus."

Today this relation between dental and ocular disease is established beyond all cavil, only in place of reflex irritation we read bacteria and their toxins. Lang considers that dental sepsis is by far the commonest cause of eye-lesions attributable to sepsis; in his opinion the uveal tract is the part most usually affected, but the sclerotic, the choroid and the retina may all be affected.

There are no doubt many in this room who could recount cases in which eye lesions have rapidly cleared up with the removal of the dental sepsis. It is a clinical fact, but the exact pathology of these eye lesions is still obscure. I have discussed the question with my ophthalmic colleagues and the majority would seem to favor a toxic origin; on the other hand, there are those who insist on an infective origin. Dr. A. W. Ormond (*Brit. Med. Journ.,* October 19, 1912, p. 1,021), for example, expresses the opinion that there is no evidence that toxins alone are capable of producing a localized inflammation of the uvea, and he considers that all intractable cases of iritis and irido-cyclitis are due to the presence of micro-organisms in the uveal tissue, and that the pathological changes produced are the result of their presence there.

In the conditions so far described the damage by the dental sepsis has been caused either by the action of toxins or by an infection of very mild intensity. Now, organisms, even though acting as saprophytes, have an unfortunate habit of suddenly acquiring a high degree of virulence, and herein lies one of the potential dangers of septic foci in the mouth.

F. St. J. Steadman* has recorded a most instructive case:

"A powerfully built man, in apparently robust health, about 40 years of age, was one day stepping off the curbstone into the street, when he suddenly became aware of a motor-'bus bearing down rapidly upon him. He sprang sharply back on to the curb, and in doing so slightly strained his left knee. The injury was so slight that he thought very little about it, and continued his usual occupation for that day. Three or four days later, however, he was admitted to Charing Cross Hospital, under the care of the late Dr. Montague Murray, suffering from acute suppurative synovitis of his knee joint. He was transferred to the surgical side. The interesting point in the case is, whence had come the infection? At the time of the accident his leg was not knocked in any way; there was no external injury, therefore, of any kind whatsoever. Dr. Montague Murray made a thorough examination of his whole body, and could find no focus of infection. He then asked me to examine his mouth, whereupon the source of the infection was immediately seen. He had a very advanced pyorrhea alveolaris; his mouth was in a filthy condition, with 'deep pockets streaming with pus around all his teeth.' The explanation given by Dr. Murray was that the 'strain' had injured some tissue cells in the immediate neighborhood of the joint, and so lowered their vitality and resistance to invasion by micro-organisms. Conse-

---

* *Trans.* 17th International Congress of Medicine, Section Stomatology, Part II, p. 146.

quently, the micro-organisms floating in the blood-stream from his oral sepsis gained a foothold, with the disastrous result that followed.''

In ulcerative endocarditis there is evidence that the source of infection may be from the teeth. Pickerill records a case where the S. salivarius was present post mortem in the bronchi, the alveoli of the lungs and in the valves of the heart, and in another case, recorded by H. Hemsted, the streptococcus obtained from the blood had the same characteristics as the S. salivarius.

In intermittent fever and that condition classified in army medical history sheets as P. U. O. (Pyrexia of unknown origin), the teeth are often to blame, and the same may be said of some cases of acute general infection.

The origin of cancer still remains a mystery, and this notwithstanding the large amount of thought and labor which has been expended upon research as to the nature of this terrible malady. In a recent issue of the *Lancet** Dr. Alex. Paine (Director of the Cancer Hospital Research Institute) contends that it ''is not a specific disease due to the activities of a special parasite, but a disordered growth of epithelium caused by various physical or chemical irritants, the most important being the toxins of micro-organisms.'' He contends that there is an intimate relation between chronic inflammatory processes and cancer, the cancer arising in the later or destructive stages of chronic inflammation and being preceded by the degeneration of the cell. From this degenerate cell, ''a cell that in some manner has been damaged or injured, the cancer cells are descended.'' ''In this light cancer appears as a terminal phase of inflammation and as a form of degeneration, in which sense indeed it was regarded by the older pathologists.'' The damage to the cell in Dr. Paine's opinion is most frequently to be found in the subtle activities of the toxins of various micro-organisms. Clinical evidence, as it

* October 2, 1920, p. 693.

accumulates, tends to support Dr. Paine's contention of the relation of chronic inflammation to cancer. In the Mayos clinic, in 153 cases of gastric carcinoma, there was naked-eye and microscopical evidence that the growth had developed in pre-existing ulcers. Cancer of the breast is frequently preceded by chronic mastitis, and I have already referred to the constant relation of chronic glossitis to cancer of the tongue.

Is there any reason to lead us to think that the toxins of dental sepsis may be a source of damage to the cell? The only work I am acquainted with that has any bearing on this subject was carried out by Mr. St. J. Steadman and reported by him to the last International Medical Congress. He examined 143 cases of cancer. The degree of dental sepsis (in these cases periodontal diseases) is indicated by figures (0) entire absence of sepsis; (1) slight sepsis; (2) moderate sepsis; (3) advanced sepsis. In these 143 cases 118 had advanced sepsis, 19 moderate, 5 slight. If we take the maximum amount of sepsis these 143 cases could have had at $3 \times 143 = 429$ we find that the amount present was 92.54 per cent. In 415 persons over 35 examined as controls, 66 had advanced, 123 moderate, 170 slight, 50 no sepsis. Taking the maximum amount of sepsis at $3 \times 415 = 1,245$ we find the amount present 614 or 49.00 per cent. These figures show that there is a greater degree of dental sepsis in patients suffering from cancer than in those free from the disease.

*From the above brief survey it will be evident that dental sepsis may affect the organisms in different ways, indeed, it may influence the course of most diseases. To quote the words of Dr. Stanley Colyer:* "The somewhat bizarre nature of the effects that have been attributed to dental sepsis may at first sight make the relationship appear improbable, but when once the principles that underlie the*

* "The Science and Practice of Dental Surgery," by Norman G. Bennett, p. 710.

*subject have been grasped, it will be realized that the conclusions are a necessary outcome. In all diseases there are always two factors at least, the seed and the soil, without which disease can not exist. The seed varies, the soil varies; never are the two the same; never is the relation repeated. This is why variation in disease is common, and in no two persons does it run a precisely similar course. Dental sepsis is but a comprehensive term to include various septic conditions in the mouth; it is not a disease. The germs causing the sepsis vary, and so the germs passing into the body and the toxin absorbed produce different results in different people. It can not be said why germs of a particular kind entering one body produce a septicæmia, and in another an infective endocarditis; or why a toxin in one will produce anæmia, and in another a neuritis; but that such is the case seems almost certain and for the time being we must be content with the fact." An explanation may be forthcoming when we understand more fully the inborn errors of metabolism and the elective affinity of organisms.*

*To us as dental practitioners dental sepsis is a constant nightmare, and when asked for an opinion on its relation to this or that condition we do not always find it easy to express our opinion in definite terms. Our first step must be to ascertain the degree of sepsis; next the possibility of other sources of sepsis must be considered. If we are able to eliminate them, then it is fair to assume that the dental sepsis is the cause, but even if other foci exist and are the probable source of infection, the fact must be remembered that dental sepsis may aid the process by lowering the general vitality.*

*It is important to place clearly before the patient the position as it appears to you and give him an idea of how far the mouth condition may be a direct or a contributing cause. I wish to lay stress on this point because at the present time rash statements are being made to patients, and they are being induced to part with their teeth on a*

*promise of complete recovery from their general condition, whatever it may be. This will lead to disappointment; dental sepsis as a pathological factor will pass under a cloud and progress be retarded.*

In discussing treatment we may consider first the cases where infection is suspected from septic pulpless teeth. I am convinced that the only way in which we can place the patient in a safe position is by removing the teeth. The X-ray picture may show no apparent trouble, but X-ray pictures are not always reliable indices of pathological conditions, and we can not rid ourselves of the fact that no technic yet devised will insure complete filling in of the dentinal tubes and the numerous canals leading from the periodontal membrane to the pulp cavity. As long as these are empty they must be regarded as areas capable of harboring sepsis. When trouble is present around the apex of the tooth I am convinced that extraction is the right course.

To discuss the treatment of sepsis arising from periodontal disease would occupy more time than is at my disposal tonight; I must therefore be content to indicate the principles which guide me in treatment. This disease, which is a progressive destruction of the tooth attachments, results in the formation of pockets around the teeth, which are filled with sepsis. The treatment is drainage; if we can efficiently drain the pockets, then the disease can be controlled, but the drainage must be thorough and must be carried out day by day by the patient. The reckless use of vaccines while neglecting this elementary principle of drainage is to be deprecated.

At the present time I think perhaps too much is expected from the removal of dental sepsis; the patient receives the impression that with extraction of teeth all will be well, but it is unreasonable to expect a long-standing gastro-intestinal condition, a septic anæmia or an arthritis, to clear up without special treatment.

I am, as you will have gathered from my remarks, a convinced believer in the harmful effects of dental sepsis. Dental sepsis is always a potential cause of disease, and we can not tell when it may become an active one. On these lines alone removal of sepsis is a wise measure of precaution. Where we must be on our guard is in giving an opinion as to its relationship to other diseases which may be present. There is a little tendency to assume that association means casual relationship, and if this is done other and more insidious conditions that are at work may be overlooked to the detriment of the patient.—*British Dental Record.*

